# Effect of dietary olive (*Olea europea*) pomace on productive performance, and physiological and meat quality parameters in Jumbo quail

**DOI:** 10.1038/s41598-023-33495-3

**Published:** 2023-04-15

**Authors:** Itumeleng S. Hlatshwayo, Caven M. Mnisi, Chidozie F. Egbu

**Affiliations:** 1 Department of Animal Science, Faculty of Natural and Agricultural Science, North-West University, Mmabatho, 2735 South Africa; 2 Food Security and Safety Focus Area, Faculty of Natural and Agricultural Sciences, North-West University, Mmabatho, South Africa; 3 Department of Agricultural Education, School of Vocation and Technical Education, Alvan Ikoku Federal College of Education, P.M.B. 1033, Owerri, Nigeria

**Keywords:** Animal biotechnology, Animal behaviour, Animal physiology

## Abstract

High levels of dietary fiber could restrict the inclusion of olive (*Olea europea* L.) pomace (OP) as a source of energy and bioactive compounds in Jumbo quail (*Coturnix* sp.) diets. In this study, the optimum inclusion level of dietary OP on growth and carcass performance, serum biochemistry, and meat quality parameters in Jumbo quail was investigated. One-week-old Jumbo quail (350; 28.9 ± 1.29 g live-weight) were reared on a standard mash grower diet with 0 (OP0), 100 (OP10), 150 (OP15), 200 (OP20), and 250 g/kg (OP25) OP for five weeks. The experimental diets were randomly allocated to 35 pens (experimental units) with seven replicates each. Overall body weight gain in Jumbo quail fed with diets OP20 and OP25 was lower (*p* < 0.001) than those fed diets OP0 and OP10. Including dietary OP had no effect on the overall gain-to-feed ratio, serum biochemistry, and internal organs but linearly reduced carcass yields. Diet OP25 promoted a higher (*p* < 0.022) meat hue angle value than the other diets. The inclusion of OP beyond 150 g/kg compromised growth and carcass performance, and altered some meat color attributes, but had no influence on serum biochemistry, and internal organs of the Jumbo quail.

## Introduction

The inclusion of agro-industrial by-products such as olive (*Olea europea* L.) pomace (OP) in animal feeds, which is rich in bioactive compounds and has a high energy value could be an environmentally friendly strategy to deliver sustainable Jumbo quail (*Coturnix* sp.) production^1^. This approach could also contribute positively towards food and nutrition security for the world’s rapidly growing human population while protecting the environment from wanton disposal of OP in landfills^2^. The OP’s high nutritional value could be of great importance to Jumbo quail production, which are birds that are known for their fast maturity, high product (meat and egg) quality, high reproductive efficiency, strong resistance to poultry diseases, high meat-to-bone ratio, and low feed and space demands^3^. Indeed, high energy and protein levels, as well as highly digestible nutrient sources are required to realize optimal quail production. However, modern poultry production systems rely on the use of maize grains and soybeans as major sources of dietary energy and protein^4^. Unfortunately, the over-reliance on these two conventional ingredients could in the future, render poultry intensification unsustainable due to price volatility, currency fluctuations, rising demands, and climatic constraints that have elevated their market prices^5^. As a result, alternatives that completely or partially replace maize or soybeans are required to reduce feeding costs, promote environmental and socio-economic sustainability, and develop profitable quail businesses^6^.

The recent increase in the demand for olive oil by the food and biofuel sectors has resulted in the generation of large quantities of OP, which is discarded in barren lands near olive processing industries. According to Adams et al.^3^, about 20% of the olive fruit is refined into extra virgin olive oil, while the remaining 80% is disposed of as olive waste. From an environmental perspective, the wanton disposal of olive waste contributes to land, soil, air, and water pollution^3,5^. Moreover, the presence of secondary metabolites such as polyphenols inhibits plant and bacterial growth, resulting in reduced biodiversity in surface water and soil^5^. Thus, the incorporation of OP in Jumbo quail feeds could be a long-term disposal management strategy, that could be adopted to enhance the sustainability of the olive oil sector^6^. Furthermore, quail producers could benefit from the OP’s low-cost complementary energy value due to high residual oil and the presence of oleuropein and hydroxytyrosol antioxidants^7^, which can potentially improve the quality of quail products. The OP contains 4.5% crude protein, 19.8% crude fat, 35.6% fiber, and 5.2% phenolics on dry matter bases^8,9^. However, the nutritional value of OP, like many other waste by-products, varies depending on the extraction method, residual oil, stage of maturity, harvesting site, and variety^10^. For this reason, it is important to investigate its feed value in Jumbo quail diets to monitor its impact on performance, and physiological and meat quality responses.

To this end, many research studies have focused on its impact as a supplemental energy source in broiler chicken diets, with conflicting results being reported. For example, dietary OP inclusion rates of up to 100 g/kg in broiler diets had no effect on weight gain^11,12^. However, the dietary inclusion of OP between 50 and 100 g/kg in the diets of broiler chicks was reported to reduce growth performance, which was linked to the high fiber content of OP^13^. According to Fadel and El-Ghonemy^14^ and Fathy et al.^15^, OP has a crude fiber content that ranges between 33 and 42.60%, which could be a major problem for simple non-ruminants such as the Jumbo quail. This is because their digestive systems cannot efficiently utilize fibrous diets, especially at an early age^3^. Thus, it is important to investigate the optimum dietary inclusion level of OP for the Jumbo quail. To date, no studies have established the maximum tolerance level of OP in the diets of Jumbo quail, which could be because it is a recently developed quail breed^3^. Thus, the aim of this study was to evaluate the effect of incremental levels of dietary OP on growth and carcass performance, serum biochemistry, visceral organs developments, and meat quality traits in Jumbo quail. It was hypothesized that the inclusion of dietary OP would improve productive performance, physiology, and meat quality traits in Jumbo quail.

## Methods

### Ethics approval

The rearing and slaughter procedure of the quail was approved by the Animal Research Ethics Committee of the North-West University (Approval no. NWU-00817-21-A5) according to established guidelines for the use and care of research animals.

### Diet formulation and chemical analyses

Fresh and wet olive pomace was acquired from Stoneberg Farm Holding (PTY) LTD (Western Cape, South Africa) and was oven-dried (60 °C) to constant weight before milling (2 mm) using a cutting mill (SM 100, Retsch GmbH, Haan, Germany). The OP chemical composition is presented in Table 1.

**Table 1:** Chemical composition (g/kg DM) of olive pomace.

Chemical composition	Proportion
Dry matter	930.0
Ash	75.00
Neutral detergent fiber	546.0
Acid detergent fiber	478.0
Fats	296.0
Crude protein	46.00
Crude fiber	240.0
Metabolizable energy (kcal/kg)	3751
Lignin	240.0
Calcium	12.50
Phosphorus	1.70
Potassium	10.50
C16:0 palmitic acid	3.60
C18:1 oleic acid	24.90
C18:2 linoleic acid	3.90
Lysine	1.00
Methionine	1.80
Phenylalanine	7.10
Threonine	7.60
Tryptophan	1.20

The other feed ingredients were purchased from Simplegrow Agric Services (Pty) Ltd and Nutroteq (Centurion, South Africa). The five isocaloric and isonitrogenous dietary treatments were formulated to satisfy the daily nutrient requirement of grower quail following guidelines from the NRC^16^. The diets were formulated (Table 2) as follows: a standard grower diet without OP (OP0) and a standard grower diet with 100 (OP10), 150 (OP15), 200 (OP20), and 250 g/kg (OP25) OP (w/w). The experimental diets were analyzed for dry matter, ash, organic matter, crude protein, ether extract, and amino acids (arginine, histidine, isoleucine, lysine, methionine, threonine, tryptophan, and valine) by methods described by the AOAC^17^. Crude fiber, nitrogen detergent fiber (NDF), and acid detergent fiber (ADF) were analyzed using the ANKOM^DELTA^ automated fiber analyzer (ANKOM Technologies, NY, USA). To evaluate the acid detergent lignin (ADL), the cellulose in the ADF residue bags was soaked in 72% H_2_SO_4_ for 3 h. The ether extract was determined using an ANKOM^XT^^15^ extractor (ANKOM Technologies, NY, USA). Minerals (calcium, chloride, phosphorus, and sodium) were analyzed using AgriLASA^18^. The metabolizable energy was calculated using the equations described by Egbu et al.^19^.

**Table 2:** Gross ingredient and chemical composition (g/kg, unless stated otherwise) of the experimental diets.

Ingredients	Diets^a^				
	OP0	OP10	OP15	OP20	OP25
Olive pomace	0.0	100.0	150.0	200.0	250.0
Yellow maize (8.5%)	519.8	421.5	372.3	323.2	274.0
Extruded full-fat soya	100.0	100.0	100.0	100.0	100.0
Soya cake (47%)	311.2	320.8	325.6	330.4	335.2
l-Methionine	1.54	1.50	1.47	1.45	1.43
Lysine-hydrochloride	0.55	0.40	0.32	0.24	0.17
Sunflower oil	36.18	27.94	23.82	19.71	15.59
Limestone	25.15	22.03	20.47	18.91	17.35
Salt-fine	1.64	1.14	0.88	0.63	0.37
Sodium bicarbonate	2.48	3.22	3.59	3.96	4.34
Br Starter PX (5 kg)	1.50	1.50	1.50	1.50	1.50

	Nutritional composition (g/kg DM)				
Dry matter	900.2	904.4	903.5	904.2	900.8
Ash	59.34	61.74	59.44	55.89	55.85
Organic matter	840.9	842.6	844.2	848.3	844.9
Crude fibre	281.3	319.0	344.0	370.6	415.7
Metabolizable energy (MJ/kg)	12.60	12.60	12.60	12.60	12.60
Crude protein	200.9	201.0	202.2	200.5	201.9
Ether extract	39.95	65.27	78.57	88.71	105.7
Neutral detergent fiber	205.6	245.4	286.8	301.1	328.8
Acid detergent fiber	121.5	132.6	142.3	152.3	161.6
Acid detergent lignin	57.30	64.26	72.11	79.09	84.54
Arginine	15.77	16.47	16.81	17.16	17.50
Histidine	6.18	6.24	6.27	6.30	6.33
Isoleucine	10.00	10.56	10.85	11.13	11.41
Lysine	13.0	13.0	13.0	13.0	13.0
Methionine	5.0	5.0	5.0	5.0	5.0
Threonine	8.75	9.29	9.56	9.83	10.10
Tryptophan	2.71	2.81	2.86	2.91	2.96
Valine	10.88	11.42	11.69	11.96	12.22
Calcium	10.0	10.0	10.0	10.0	10.0
Chloride	1.40	1.40	1.40	1.40	1.40
Phosphorus	3.05	3.05	3.05	3.05	3.05
Sodium	1.50	1.50	1.50	1.50	1.50

^a^Diets: *OP0* a standard grower diet without olive pomace, *OP10* a standard grower diet with 100 g/kg olive pomace, *OP15* a standard grower diet with 150 g/kg olive pomace, *OP20* a standard grower diet with 200 g/kg olive pomace, *OP25* a standard grower diet with 250 g/kg olive pomace.

### Experimental design and feeding trial

The feeding experiment was carried out in December 2021 at the North-West University Research Farm (25°86′00″ S; 25°64′32″ E), South Africa. A total of 350, 4-day-old Jumbo quail chicks were purchased from Golden Quail Farm (Randfontein, South Africa). In a completely randomized design, the chicks were evenly allocated into 35 replicate pens, to which the five experimental diets were randomly distributed producing 7 replicates per dietary treatment. The pens (100 cm Length × 60 cm Width × 30 cm Height) were considered as the experimental units, each holding 10 birds. The chicks were orally dosed with vitamins and electrolytes for 3 days to offset stress and adapted to the pens and experimental diets until day 7 of age. Measurements commenced from 7 to 42 days of age, and rearing was conducted under natural lighting (12 h of day length). The diets and clean water were offered ad libitum during the feeding trial. Initial live weights (28.9 ± 1.29 g) were recorded and subsequently weighed weekly per pen to determine average weekly body weight gain. Feed intake was determined from the amount of feed offered the previous day and the refusals received the following morning. Gain-to-feed ratio (G:F) was determined by dividing weight gain by feed consumption data. The mortality rates were considered in the calculation of the growth performance data.

### Slaughter procedure and blood collection

On day 42 of age, all the birds per pen (experimental unit) were weighed to determine their final body weight. The birds were then transported to a nearby poultry abattoir where they were all stunned and then slaughtered after resting for 2 h. About 2 mL of fresh blood was randomly collected from two quail per experimental unit whose jugular veins had been sliced with a sharp knife. The blood was immediately transferred into red-top tubes and left standing at room temperature to allow the recovery of serum from clotted blood. The blood samples were then centrifuged for 10 min at 3500 rpm using a Centrifuge (model Z 206 A, Hermle Labortechnik GmbH, Germany) supplied by Lasec SA (Pty) Ltd (Midrand, South Africa). The following serum biochemical parameters: alanine transaminase (ALT), albumin, amylase, calcium, cholesterol, creatine, glucose, symmetric dimethylarginine (SDMA), total bilirubin, total protein, and urea were analyzed using an automated Vet Test Chemistry Analyzer from IDEXX Laboratories (Midrand, South Africa).

### Carcass, visceral organs, and meat quality

The carcasses were weighed immediately after dressing and evisceration to obtain hot carcass weight (HCW) and then chilled at 16 °C for 24 h to determine cold carcass weight (CCW). The carcasses were then cut to measure (Explorer® EX224, OHAUS Corp, US) the wings, drumstick, thigh, and breast weights. Carcass yield was calculated as a proportion of HCW to final body weight. The weights of the gizzard, liver, spleen, proventriculus, small intestines, and ceca with their contents were measured using the digital scale described above. A measuring tape was used to determine the small intestines, ceca, and colon lengths. Breast pH was measured 1 h and 24 h post-mortem using a Corning Model 4 pH-temperature meter (Corning Glass Works, Medfield, MA, USA), which was calibrated using standard pH solutions after measuring each experimental unit^20^. Breast color coordinates (L* = brightness, a* = redness, and b* = yellowness) were determined using a color spectrophotometer (CM 2500c, Konika Minolta, Osaka, Japan) 1 h and 24 h post-mortem^20^. The filter-paper press technique by Whiting and Jenkins^21^ was used to determine breast meat water holding capacity (WHC). Honikel^22^ techniques were used to measure breast cooking loss.

### Statistical analysis

Weekly measured data (feed intake, weight gain, and gain-to-feed ratio) were analyzed using the repeated measures analysis in the procedure of general linear model (PROC GLM) in SAS^23^ to assess the interaction effects of diet and time (quail age in weeks). The linear and quadratic coefficients for all the measured parameters were evaluated using polynomial contrasts by means of response surface regression (PROC RSREG) in SAS^23^. Dietary differences among the parameters were also analyzed using one-way ANOVA by means of PROC GLM^23^. The level of significance was set at *p* < 0.05 and the probability of difference option in SAS was used to separate the treatment means.

### Animal welfare statement

The authors confirm that the ethical policies of the journal, as noted on the journal's author guidelines page, have been adhered to and the appropriate ethical review committee approval has been received. The authors confirm that they have followed EU standards for the protection of animals used for scientific purposes.

The authors confirm that all methods were carried out in accordance with relevant guidelines and regulations.

### Research involving plants

The collection of Olive (*Olea europea*) pomace complies with relevant institutional, national, and international guidelines and legislation as recommended by the IUCN Policy Statement on Research Involving Species at Risk of Extinction and the Convention on the Trade in Endangered Species of Wild Fauna and Flora.

The fresh and wet Olive (*Olea europea*) pomace used in the study was bought from Stoneberg Farm Holding (PTY) LTD (Western Cape, South Africa).

### Research involving animals

The study was reported in accordance with ARRIVE guidelines.

## Results

### Growth performance

Repeated measures analysis revealed a significant week × diet interaction effect on average weekly feed intake (AWFI, *p* = 0.003) but no interaction effects were observed on body weight gain (*p* = 0.335) and G:F (*p* = 0.063). Table 3 shows that the inclusion of incremental levels of dietary OP resulted in linear decreases in feed intake in week 3 [*y* = 67.2 (± 2.44)–0.547 (± 0.709) *x*; R^2^ = 0.558, *p* = 0.021] and week 4 [*y* = 75.1 (± 2.58)–0.003 (± 0.752) *x*; R^2^ = 0.723, *p* = 0.002]. The GLM results showed that there were significant dietary effects from week 2 to week 6. Two-week-old quail reared on diet OP25 had the lowest feed intake followed by those reared on diet OP20 and the highest was from those on the OP0, OP10, and OP15 groups, whose feed intake did not differ (*p* > 0.05). Three-week-old quail fed with diets OP20 and OP25 had lower feed intake than those reared on diets OP0, OP10, and OP15, which were statistically similar (*p* > 0.05). In week 4, diet OP20 caused lower feed intake than diets OP0 and OP10, which statistically did not vary from diets OP15 and OP25. In week 5, diet OP25 promoted lower feed intake than diets OP0, OP10, and OP20, which did not differ (*p* > 0.05). Six-week-old quail on OP10 treatment had the highest feed intake (84.35 g/bird) and the lowest feed intake was observed from birds on OP25 treatment (39.81 g/bird).

**Table 3:** Effects of incremental levels of dietary olive pomace on growth performance in Jumbo quail.

	Diets^1^					SEM	Significance		
	OP0	OP10	OP15	OP20	OP25		*P*-_GLM_	*P*-_Linear_	*P*-_Quadratic_
	Average feed intake								
Week 2	53.65^a^	55.53^a^	53.47^a^	45.92^b^	38.85^c^	1.513	< 0.0001	0.200	0.419
Week 3	65.61^a^	62.24^a^	61.11^a^	50.83^b^	46.97^b^	2.461	< 0.0001	0.021	0.970
Week 4	73.65^a^	70.42^a^	62.17^ab^	52.17^b^	64.22^a,b^	3.541	0.002	0.002	0.213
Week 5	82.42^a^	78.67^a^	67.75^a,b^	70.03^a^	47.37^b^	5.395	0.001	0.117	0.456
Week 6	73.54^b^	84.35^a^	71.29^c^	65.48^d^	39.81^e^	0.018	0.002	0.292	0.187
Overall BWG	88.83^a^	84.99^a^	77.89^b^	57.63^c^	53.45^c^	5.68	0.001	0.035	0.136
Overall G:F	0.253	0.242	0.251	0.209	0.223	0.018	0.350	0.070	0.064

^a–^^e^In a row, means with common superscripts do not differ (*p* < 0.05).

^1^Diets: *OP0* a standard grower diet without olive pomace, *OP10* a standard grower diet with 100 g/kg olive pomace, *OP15* a standard grower diet with 150 g/kg olive pomace, *OP20* a standard grower diet with 200 g/kg olive pomace, *OP25* a standard grower diet with 250 g/kg olive pomace.

The regression results showed that overall BWG linearly decreased [*y* = 126 (± 2.58)–1.673 (± 1.909)* x*; R^2^ = 0.408, *p* = 0.036] as dietary OP levels increased. Likewise, dietary differences were observed in overall BWG, where quail reared on diets OP20 and OP25 recorded the lowest (*p* < 0.05) weight gain followed by those on diet OP15, and the highest weight gain was from those on diets OP0 and OP10.

Neither linear nor quadratic effects (*p* > 0.05) were recorded for overall G:F as dietary OP levels increased. Similarly, no significant differences were observed among the treatment groups on overall G:F.

### Serum biochemistry

Regression results showed that there were no significant linear or quadratic effects (*p* > 0.05) for all serum biochemical parameters as dietary OP levels increased (Table 4). The inclusion of dietary OP also induced no significant dietary effects on all serum biochemical parameters of the Jumbo quail.

**Table 4:** Effects of incremental levels of dietary olive pomace on serum biochemical parameters in Jumbo quail.

Parameters^2^	Diets^1^					SEM	Significance	
	OP0	OP10	OP15	OP20	OP25		*P*-_GLM_	*P*-_Linear_
Albumin (g/L)	22.88	42.57	33.60	25.63	31.83	8.996	0.195	0.811
ALT (U/L)	49.06	73.38	38.00	96.61	59.83	22.55	0.284	0.389
Amylase (U/L)	260.4	299.8	243.0	250.0	185.5	62.57	0.481	0.150
Calcium (mmol/L)	1.44	1.40	1.42	1.48	1.69	0.213	0.839	0.244
Cholesterol (mmol/L)	4.06	4.10	3.58	4.34	2.84	1.010	0.717	0.532
Creatinine (µmol/L)	10.44	9.43	9.30	9.00	9.00	1.410	0.839	0.374
Glucose (mmol/L)	5.16	3.69	4.32	3.07	2.80	0.872	0.204	0.380
SDMA (µg/dL)	18.75	6.25	28.33	13.00	5.25	9.624	0.180	0.380
Total bilirubin (µmol/L)	91.00	160.1	128.3	79.13	109.5	9.754	0.898	0.816
Total protein (g/L)	59.71	78.30	66.67	49.75	82.50	32.25	0.777	0.346
Urea (mmol/L)	1.89	2.70	1.85	3.05	1.72	0.842	0.591	0.670

^1^Diets: *OP0* a standard grower diet without olive pomace, *OP10* a standard grower diet with 100 g/kg olive pomace, *OP15* a standard grower diet with 150 g/kg olive pomace, *OP20* a standard grower diet with 200 g/kg olive pomace, *OP25* a standard grower diet with 250 g/kg olive pomace.

^2^Parameters: *ALT* alanine transaminase, *SDMA* symmetric dimethylarginine.

### Carcass performance and internal organs

Table 5 shows that the inclusion of increasing dietary OP levels linearly reduced carcass yield [*y* = 65.57 (± 2.55)–0.15 (± 0.48)* x*; R^2^ = 0.167, *p* = 0.040], HCW [*y* = 78.99 (± 4.53)–0.55 (± 0.87) *x*; R^2^ = 0.395, *p* = 0.002] and CCW [*y* = 73.9 (± 4.10)–0.066 (± 0.779) *x*; R^2^ = 0.416, *p* = 0.0003]. Likewise, linear decreases were recorded for drumstick [*y* = 3.85 (± 0.23)–0.03 (± 0.04) *x*; R^2^ = 0.262, *p* = 0.007], thigh [*y* = 4.69 (± 0.37)–0.01 (± 0.07) *x*; R^2^ = 0.262, *p* = 0.008], wing [*y* = 4.84 (± 0.24)–0.04 (± 0.04) *x*; R^2^ = 0.386, *p* = 0.001], and breast weights [*y* = 11.60 (± 1.08)–0.03 (± 0.20) *x*; R^2^ = 0.165, *p* = 0.042]. However, neither linear nor quadratic (*p* > 0.05) effects were recorded for all the visceral organs as dietary OP levels were increased. The dietary treatments had significant effects on HCW, CCW, and wing weight only. Quail reared on diet OP25 had lighter (*p* < 0.05) HCW and wings than those on diet OP0, whose HCW and wings were statistically similar (*p* > 0.05) to those reared on diets OP10, OP15, and OP20. The control diet OP0 promoted the heaviest (*p* < 0.05) CCW (74.0 g) while the OP25 treatment resulted in the lightest CCW (47.3 g).

**Table 5:** Effects of incremental levels of dietary olive pomace on carcass performance and organ development (g, unless stated otherwise) in Jumbo quail.

Parameters^2^	Diets^1^					SEM	Significance		
	OP0	OP10	OP15	OP20	OP25		*P-*_*GLM*_	*P-*_*Linear*_	*P-*_*Quadratic*_
Carcass yield (%)	65.2	64.3	63.8	54.8	59.9	3.274	0.090	0.040	0.712
HCW	77.0^a^	70.5^a,b^	69.3^a,b^	56.8^a,b^	49.5^b^	6.618	0.018	0.002	0.560
CCW	74.0^a^	68.7^a,b^	65.8^a,b^	54.4^b,c^	47.3^c^	4.314	0.009	0.0003	0.207
Drumstick	3.89	3.37	3.40	3.30	2.68	0.310	0.070	0.007	0.766
Thigh	4.67	4.44	3.84	3.66	3.05	0.507	0.110	0.008	0.461
Wing	4.85^a^	4.32^a,b^	3.93^a,b^	4.04^a,b^	3.30^b^	0.320	0.011	0.001	0.803
Breast	11.9	11.2	9.06	10.1	7.65	1.465	0.227	0.042	0.582
Gizzard	2.33	2.39	2.42	2.53	2.25	0.224	0.906	0.390	0.840
Liver	2.51	2.53	2.33	2.52	2.53	0.313	0.983	0.227	0.296
Proventriculus	0.60	0.62	0.57	0.90	0.49	0.177	0.449	0.495	0.963
Spleen	0.17	0.12	0.10	0.54	0.05	0.249	0.430	0.525	0.789
Ceca	1.24	1.47	1.25	1.57	1.14	0.182	0.313	0.675	0.150
Small intestine	4.45	4.69	4.41	4.32	3.63	0.446	0.457	0.374	0.210
Ceca (cm)	9.64	10.2	9.57	8.29	8.20	0.872	0.273	0.080	0.077
Colon (cm)	4.74	4.63	4.18	2.95	4.83	0.836	0.250	0.360	0.522
Small intestine (cm)	61.0	62.5	63.4	47.7	57.0	5.858	0.217	0.166	0.286

^a–^^c^In a row, means with common superscripts do not differ (*p* < 0.05).

^1^Diets: *OP0* a standard grower diet without olive pomace, *OP10* a standard grower diet with 100 g/kg olive pomace, *OP15* a standard grower diet with 150 g/kg olive pomace, *OP20* a standard grower diet with 200 g/kg olive pomace, *OP25* a standard grower diet mixed with 250 g/kg olive pomace.

^2^Parameters: *HCW* hot carcass weight, *CCW* cold carcass weight.

### Meat quality

Table 6 shows that there were linear increases for 1-h breast meat yellowness (b^*^_1_) [*y* = 2.85 (± 0.55) + 0.014 (± 0.104) *x*; R^2^ = 0.270, *p* = 0.009] and 1-h hue angle [*y* = 0.39 (± 0.06) + 0.01 (± 0.01) *x*; R^2^ = 0.248, *p* = 0.010]. However, linear decreases were recorded for 24-h hue angle [*y* = 0.56 (± 0.08)–0.01 (± 0.01) *x*; R^2^ = 0.318, *p* = 0.006] and 24-h redness (a^*^_24_) [*y* = 6.81 (± 0.24)–0.11 (± 0.04) *x*; R^2^ = 0.256, *p* = 0.036] as dietary OP levels were increased. The dietary treatments had significant effects on a^*^_1_, b^*^_1_, chroma_1_, and hue angle_24_. Birds reared on diet OP25 had higher (*p* < 0.05) a*_1_ than those on diet OP10, but did not differ from those reared on OP0, OP15, and OP20. Quail reared on diet OP25 had higher b^*^_1_ than those on diets OP15 and OP20, whose b^*^_1_ were statistically similar (*p* > 0.05) to those reared on diets OP0 and OP10. Diet OP10 promoted lower chroma_1_ values than diets OP15, and OP20, which did not differ (*p* > 0.05). Quail reared on diet OP25 had higher meat hue Angle_24_ values than those reared on diet OP10, but statistically did not differ from those reared on OP0, OP15, and OP20 which did not vary.

**Table 6:** Effects of incremental levels of dietary olive pomace on meat quality parameters in Jumbo quail.

Parameters^2^	Diets^1^					SEM	Significance		
	OP0	OP10	OP15	OP20	OP25		*P-*_*GLM*_	*P-*_*Linear*_	*P-*_*Quadratic*_
pH_1_	5.84	5.91	6.02	6.04	6.00	0.080	0.460	0.076	0.715
L*_1_ (lightness)	53.17	53.06	49.44	55.11	54.02	2.104	0.590	0.824	0.446
a*_1_ (redness)	6.74^a,b^	5.70^b^	6.97^a,b^	6.21^a,b^	7.31^a^	0.320	0.044	0.660	0.056
b*_1_ (yellowness)	2.97^b^	2.71^b^	4.80^a,b^	4.54^a,b^	5.19^a^	0.535	0.029	0.009	0.416
Chroma_1_	7.61^a,b^	6.50^b^	8.55^a^	7.82^a,b^	8.98^a^	0.428	0.020	0.105	0.088
Hue angle_1_	0.40	0.43	0.61	0.61	0.61	0.061	0.077	0.010	0.921
pH_24_	6.06	6.06	6.16	6.13	6.26	0.126	0.693	0.210	0.570
L*_24_ (lightness)	50.11	51.37	53.62	53.28	51.38	2.300	0.677	0.306	0.419
a*_24_ (redness)	6.79	6.12	5.93	5.74	6.31	0.328	0.094	0.036	0.067
b*_24_ (Yellowness)	4.37	3.61	5.67	5.18	54.93	17.84	0.182	0.115	0.109
Chroma_24_	8.22	7.27	8.31	7.85	57.07	17.59	0.190	0.131	0.108
Hue angle_24_	0.57^a,b^	0.51^b^	0.75^a,b^	0.72^a,b^	0.93^a^	0.101	0.022	0.006	0.125
Cooking loss (%)	50.29	53.26	48.30	52.95	42.88	3.20	0.528	0.627	0.310
WHC (%)	89.97	89.68	89.81	88.77	90.11	1.99	0.992	0.565	0.100

^a,b^In a row, means with common superscripts do not differ (*p* < 0.05).

^1^Diets: *OP0* a standard grower diet without olive pomace, *OP10* a standard grower diet with 100 g/kg olive pomace, *OP15* a standard grower diet with 150 g/kg olive pomace, *OP20* a standard grower diet with 200 g/kg olive pomace, *OP25* a standard grower diet with 250 g/kg olive pomace.

^2^Parameters: *pH*_*1*_ pH 1-h post-mortem, *pH*_*24*_ pH 24-h post-mortem, *WHC* water holding capacity.

## Discussion

The adoption of agro-waste by-products as sources of nutrients and bioactive compounds is crucial in ensuring long-term intensive quail production while reducing environmental pollution^24^. Olive pomace is one such waste by-product that can be used to supply dietary energy and phytochemicals to the quail subsector Sayehban et al.^13^ and as such, provide an innovative and efficient strategy for recycling. To this end, no studies have evaluated the effect of OP inclusion in Jumbo quail diets, thus the current study investigated the optimum inclusion level of dietary OP in diets of Jumbo quail.

The results showed a significant diet × week interaction effect on feed intake, which demonstrates that the capacity of the birds to consume the dietary treatments varied with age. Including dietary OP up to 150 g/kg promoted similar feed consumption as the control diet, however, levels up to 250 g/kg linearly reduced feed consumption. This indicates that higher levels of dietary OP suppress feed utilization, which could be due to high fiber (24 g/kg DM) levels in OP. This finding agrees with the results of Saleh and Alzawqari^25^ who noted reduced feed intake when 200 g/kg olive cake meal was used to replace corn in broiler diets. Similarly, the diets containing OP at 200–250 g/kg promoted the lowest overall weight gain than those containing 0–100 g/kg OP, further confirming that the antinutritional effects of fiber in OP might have reduced nutrient absorption into body mass. The lack of adverse effects on feed intake and weight gain when OP was included up to 150 g/kg corroborates the findings of Sayehban et al.^13^, who reported that OP levels as high as 100 g/kg can be included in broiler diets without detrimental effects on performance. There were no dietary effects on overall G:F, which demonstrates that the inclusion of OP in the diets did not enhance the birds’ capacity to efficiently convert the feed into body mass. Numerically, birds in the control group had higher G:F values than those in the OP treatments, which confirms that the inclusion of OP in Jumbo quail diets compromised growth performance. Contrary to the results, Pappas et al.^26^ reported that the supplementation of a broiler finisher diet with OP at 80 g/kg resulted in poorer G:F than the control. The authors opined that the fiber content of OP was responsible for the poor G:F. The reported poor growth performance across research might be attributed to changes in nutrient composition, particularly the insoluble fiber and condensed tannin content of the olive by-products utilized.

Serum biochemical parameters provide an efficient evaluation of any pathophysiological aberrations in animals^27^. All serum biochemical indices showed no diet-induced variations as OP inclusion levels increased. These results disagree with previous studies that reported a decrease in serum cholesterol when an olive cake meal was used to replace maize at 100 g/kg in a broiler diet^12,25^. Since dietary OP contains antinutrients such as condensed tannins and insoluble fibers, it was hypothesized that dietary inclusion of OP would have a deleterious influence on serum biochemical parameters. Also, the lack of dietary effects on serum biochemical parameters measured could be because the diets were isonitrogenous and isocaloric. Albumin is a protein present in blood plasma, and low amounts indicate renal malfunction, liver damage, inflammation, or infections, whereas high levels suggest dehydration or severe diarrhea. Furthermore, serum total protein was similar which could be expected since the dietary treatments on analyses contained similar numerical crude protein values. Also, the liver enzyme (ALT) was unaltered, indicating that the birds did not suffer from diet-induced hepatotoxicity. Nonetheless, all the serum biochemical indices were within the normal ranges reported for a healthy quail^28–30^.

Variations in dietary fiber content have been linked to changes in the gastrointestinal morphology of poultry as an adaptive mechanism to utilize the high fiber levels^31^. Surprisingly, no dietary effects were recorded on all the examined visceral organs despite high levels of insoluble fiber in the OP-containing treatments. Similarly, Sayehban et al.^13^ observed no differences in the visceral organs of broilers supplemented with 100 g/kg olive pulp in their diets. It is probable that levels up to 250 g/kg of dietary OP were insufficient to induce physio-anatomical alterations in visceral organs. However, growth parameters were negatively impacted at 250 g/kg of dietary OP inclusion, indicating that there is a need to valorize OP with exogenous fibrolytic enzymes and/or polyethylene glycol to enable larger amounts of OP inclusion in Jumbo quail diets.

The viability and profitability of poultry production have been strongly linked to carcass yield enhancements. Understanding the elements that influence carcass characteristics (breasts, wings, thighs, and drumsticks) is thus critical for the success of any poultry business. Unfortunately, there is a limited understanding of such parameters in quail birds^32^_,_ which could be because quail birds are mostly sold as live birds or whole carcasses. In this study, the inclusion of OP linearly decreased carcass yield, HCW, CCW, and weights of drumsticks, thigh, wing, and breast. This observation could be linked to the reduced feed intake observed among the OP-containing diets, which resulted in poor nutrient uptake for muscle development leading to poor weight gains. This indicates that the use of OP in quail diets could reduce the profitability and economic sustainability of quail enterprises. Furthermore, these results are inconsistent with the findings of Saleh and Alzawqari^25^ as well as Pappas et al.^26^, who recorded no significant differences on carcass characteristics of broilers when 80 and 100 g/kg of olive waste by-products were used to replace maize in diets.

Meat color, water holding capacity (WHC), cook losses, texture, and sensory properties all contribute to overall customer acceptance of meat and meat products^33^. Color differences from the expected norm on retail displays cause consumers to reject meat products. Color differences were found in all treatment groups, indicating that the inclusion of OP positively influenced the appearance of the meat. A linear increase was recorded for meat yellowness and hue angle measured 1 h post-mortem which could be due to the presence of carotene and other pigments in olive residues^34^ that are known to improve meat yellowness. Furthermore, meat redness and hue angle measured 24 h post-mortem linearly declined, which could be attributable to the exposure of the meat to oxygen upon storage that triggers oxidative metabolism, and as such, produces free radical by-products that ultimately cause myoglobin oxidation resulting in brown pigment metmyoglobin^35^. Meat pH is mostly associated with the biochemistry condition of the muscle at the point of slaughter, which causes rigor mortis to occur^36^. Chan et al.^37^ identified a link between meat pH and color, with lighter meat indicating a low pH value and dark meat indicating a high pH value. Nonetheless, the dietary treatments in this study had no influence on meat pH_1_ (5.84–6.04) and pH_24_ (6.06–6.26), which was within the normal meat pH (5.7–6.5) range reported by Mulaudzi et al.^32^. The ability of meat to retain water when subjected to external forces such as slicing, grinding, and compressing is referred to as WHC^38^. The amount of water lost or retained is mostly determined by the pH of the tissue; for instance, higher pH results in less water loss, whereas a lower pH value determines the amount of water retained by the meat^39^. When meat pH is low, protein denaturation occurs, which further lowers the meat's WHC^40^. The lack of dietary variations in meat pH may explain why the meat exhibited non-significant WHC values. Indeed, no variations in the cooking loss were identified, suggesting that OP supplementation did not impair the organoleptic (juiciness, tenderness, texture, smell, and color) and sensory aspects^41,42^ of quail meat.

## Conclusion

Dietary olive pomace above 150 g/kg compromised growth and carcass performance, but not serum biochemical and meat quality parameters of the Jumbo quail. It is recommended that where higher dietary levels of the pomace are desired, fiber-degrading strategies can be employed prior to its inclusion in Jumbo quail diets.

## Data Availability

The data supporting this study’s findings are available from the corresponding author upon reasonable request.
